# The Antitumor Activities of *Marsdenia tenacissima*

**DOI:** 10.3389/fonc.2018.00473

**Published:** 2018-10-23

**Authors:** Xiang Wang, Yuanliang Yan, Xi Chen, Shuangshuang Zeng, Long Qian, Xinxin Ren, Jie Wei, Xue Yang, Yangying Zhou, Zhicheng Gong, Zhijie Xu

**Affiliations:** ^1^Department of Pharmacy, Xiangya Hospital, Central South University, Changsha, China; ^2^National Clinical Research Center for Geriatric Disorders, Xiangya Hospital, Central South University, Changsha, China; ^3^Key Laboratory of Molecular Radiation Oncology of Hunan Province, Center for Molecular Medicine, Xiangya Hospital, Central South University, Changsha, China; ^4^Department of Oncology, Xiangya Hospital, Central South University, Changsha, China; ^5^Department of Pathology, Xiangya Hospital, Central South University, Changsha, China

**Keywords:** *Marsdenia tenacissima*, antitumor activities, malignant tumor, signaling pathways, efficacy, toxicity

## Abstract

*Marsdenia tenacissima* (MT), a traditional Chinese herbal medicine, has long been used for thousands of years to treat asthma, tracheitis, rheumatism, etc. An increasing number of recent studies have focused on the antitumor effects of MT. The effects of MT on cancer are the result of various activated signaling pathways and inhibiting factors and the high expression levels of regulatory proteins. MT can inhibit different cancer types including non-small cell lung cancer (NSCLC), malignant tumors, hepatic carcinoma, and so on. This article mainly focuses on the activities and mechanisms of MT. In addition, the efficacy and toxicity of MT are also discussed. Further studies of MT are required for improved medicinal utilization.

## Introduction

Cancer is a growing class of diseases that could influence any part of the body and is becoming the primary cause of death in the world (1, 2). Radiation, surgery and drugs are effective approaches for the treatment of cancer. Chemotherapy drugs can improve the quality life of cancer patients, but drug resistance and severe adverse side effects, such as damage to liver function, bone marrow suppression, and neurotoxicity, are significant obstacles that decrease treatment-related tolerance and compliance leading to therapeutic failure (3, 4). Thus, there is an urgent need to develop novel drugs that have the advantages of being more effective, causing fewer side effects and simultaneously overcoming drug resistance in a variety of cancers. In recent years, there has been increased interest in the natural products from plants that play important roles in research and development of therapeutics agents for cancer. For example, many antitumor drugs in clinical used, such as podophyllotoxins (5), diosmetins (6), and taxanes (7) are taken from plants.

*Marsdenia tenacissima* (Family Asclepiadaceae) is a perennial climber that is extensive distributed in tropical to subtropical areas in Asia, primarily in the Yunnan and Guizhou Provinces of China (8). The dried stems of *Marsdenia tenacissima* (MT), known as “Tong-guang-san” or “Tong-guang-teng,” recorded in the 2010 edition of “pharmacopeia of the People's Republic of China” (9), have been used in Chinese folk medicine for thousands of years. The medicinal use of this plant can be traced back to the Ming Dynasty and is primarily recorded in “Dian Nan Ben Cao” by Mao Lan (1397–1470) (10). MT, with a bitter taste and slightly cold, has a wide range of biological activities, including asthma and cough relief, anti-inflammatory and anticancer functions (11). Modern pharmacological studies have revealed that MT has obvious antitumor, hepato protective, diuretic, and immunomodulatory effects and it has a specific effect on various tumors including ovarian cancer (12), liver cancer (13), adenoid cystic carcinoma (14), etc. *Marsdenia tenacissima* extract (MTE, trade name: Xiao-Ai-Ping injection) improves quality of life, strengthens immune function and effectively prolongs the viable period of cancer patients (15). Xiao-Ai-Ping (XAP) injection, of which the main constituent is MT, has been authorized in the Chinese market for decades to be used alone or combined with radiotherapy or chemotherapy for cancer treatment (16).

**Figure 1:**
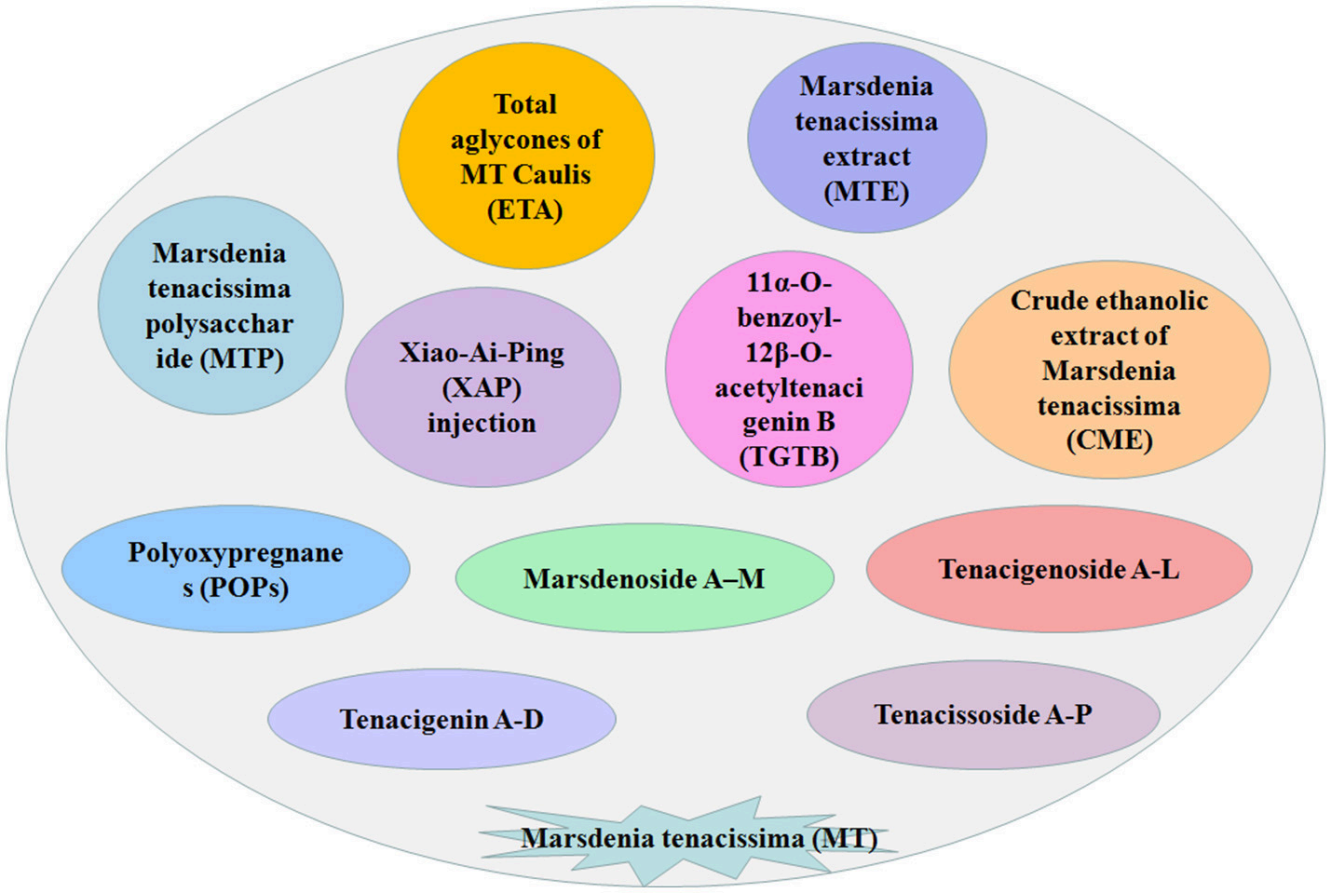
The substances associated with *Marsdenia tenacissima*. The main components of the Xiao-Ai-Ping (XAP) injection is *Marsdenia tenacissima* (MT).

So far, more than 50 C21 steroidal glycosides have been isolated and identified from MT (Figure 1, Table 1), including tenacissoside A–P, marsdenoside A–M, tenacigenoside A–L, tenacigenin A–D, and their derivatives (18, 32, 33). Most are derived from tenacigenin B, the steroidal skeleton of polyoxypregnane (POP) glycosides (8). The differences in their chemical structure are the various substituted groups connected to C-11 and/ or C-12, acylated groups such as acetyl, 2-methylbutyryl and benzoyl (18). The major bioactive constituents of C21 steroids are POP glycosides or aglycones and these are abundant in the stems of MT. Studies have found that C21 steroidal glycosides, the substances isolated from plants, have various effects such as antitumor, immunomodulatory, and liver protection (14). MT contains steroidal glycosides, polysaccharides, organic acids, and other chemical components, of which C21 steroidal glycosides are known as the major antitumor substances via multiple mechanisms, such as disrupting cancer cell proliferation and metastasis, regulating signaling pathways, and reversing multidrug resistance (18, 34).

**Table 1:** The compounds isolated from herb medicine MT and their anti-tumor activities *in vivo* and *in vitro*.

**Full names**	**Abbreviation**	**Formulation**	**Administration**	**Cell lines /Animals**	**Cancers**	**Modulated factors**	**Biological effects**	**Drug concentration**	**References**
*Marsdenia tenacissima* extract	MTE	Compound		HCC827/ER cells	Lung cancer	Axl, c-Met	Reversing multidrug resistance	0.5–500 mg/ml	(17)
			Orally administration	HCC827/ER xenograft nude mice		EGFR		5 g/kg	
Tenacigenin D		Monomer		H460/ H460- Vbl cells		K-Ras, P-gp		10, 20 μM	(18)
*Marsdenia tenacissima* extract	MTE	Compound	Injection	H460 xenografts nude mice		PI3K/AKT/mTOR ERK1/2	Inducing apoptosis	5 g/kg	(19)
			Injection	H1975 xenografts nude mice			Inhibiting proliferation, inducing apoptosis	5 g/kg	(20)
				H460,H1975 cells				0.5–500 μl/ml	
				HCC827 cells				8 mg/ml	(21)
*Marsdenia tenacissima* extract	MTE	Compound		Jurkat cells	Hematological Malignancy	PI3K/AKT/mTOR	Inhibiting proliferation, inducing apoptosis	0–640 μg/ml	(22)
			Intraperitoneal injection	A20 lymphoma cells		MMP-2, MMP-9	Inhibiting growth, decreasing angiogenesis	0–50 μl/ml	(23)
Crude ethanolic extract	CME	Compound	Intraperitoneal injection	K562 xenografts nude mice		Bax, caspase-9, caspase-3,	Inducing apoptosis	20–320 μg/mL	(3)
				K562 cells		Cyclin D1	Promoting G0/G1 arrest		
Tenacigeno side A, 11α-O-benz oyl-12β-O-acetyl tenacigenin B	TGTA, TGTB	Monomer		Raji lymphoma cells		BCL2,BCL-XL,BID	Inducing apoptosis	1, 10, 50 μL/mL	(24)
			Intraperitoneal injection	lymphoma bearing NOD/SCID nude mice				25 mg/kg	
Tenacisso side C	TGTC	Monomer		K562 cells		Bcl-2,Bcl-xL, Bax,Bak, caspase-9, caspase-3	Inducing apoptosis	0–40 μM	(25)
			Injected subcutaneously	K562 bearing nude mice		cycline D1	Promoting G0/G1 arrest	8, 16 mg/kg	
*Marsdenia tenacissima*	MT	Compound		HepG2 cells	hepatic carcinoma	CYP2D6, CYP3A4		10 mg/ml	(26)
				HepG2 cells		VEGF-A	Decreasing angiogenesis	0, 2.5, 5, 7.5 mg/ml	(27)
*Marsdenia tenacissima* polysaccharide	MTP	Compound	Intragastrically	H22 tumor-bearing mice		GSH-Px,SOD,CAT	Enhancing immune, inhibiting tumor growth	50, 100, 200 mg/kg	(11)
Five main C21 steroids	FR5	Compound		HepG2,Bel7402 cells		Hippo, PI3K-PTEN-mTOR	Inhibiting proliferation, inducing apoptosis	80, 160 g/ml	(28)
Tenacissimo side A, 11α-O-benz oyl-12β-O-acetyl tenacigenin B	TGTA, TGTB	Monomer		HepG2/Dox Cells		P-gp	Reversing multidrug resistance	20 μg/mL	(29)
*Marsdenia tenacissima*	MT	Compound		KYSE150, Eca-109 cells	Esophageal cancer	MAPK	Inhibiting proliferation	0–200 mg/ml	(9)
*Marsdenia tenacissima*	MT	Compound		MG63 cells	Osteosarcoma	Fas	Sensibilization	50 mg/mL	(30)
Total aglycones of MT	ETA	Compound	Oral administration	KB-3-1 tumor bearing mice	Oral epidermoid carcinoma	P-gp	Sensibilization	250 mg/kg	(31)
Polyoxypregnanes	POPs	Compound		SW620/Ad300, MCF-7/VP, MCF-7/FLV1000 cells	Colon carcinoma, Breast cancer	P-gp, MRP1, BCRP	Reversing multidrug resistance	0.4, 2, 10 μM	(10)

**Figure 2:**
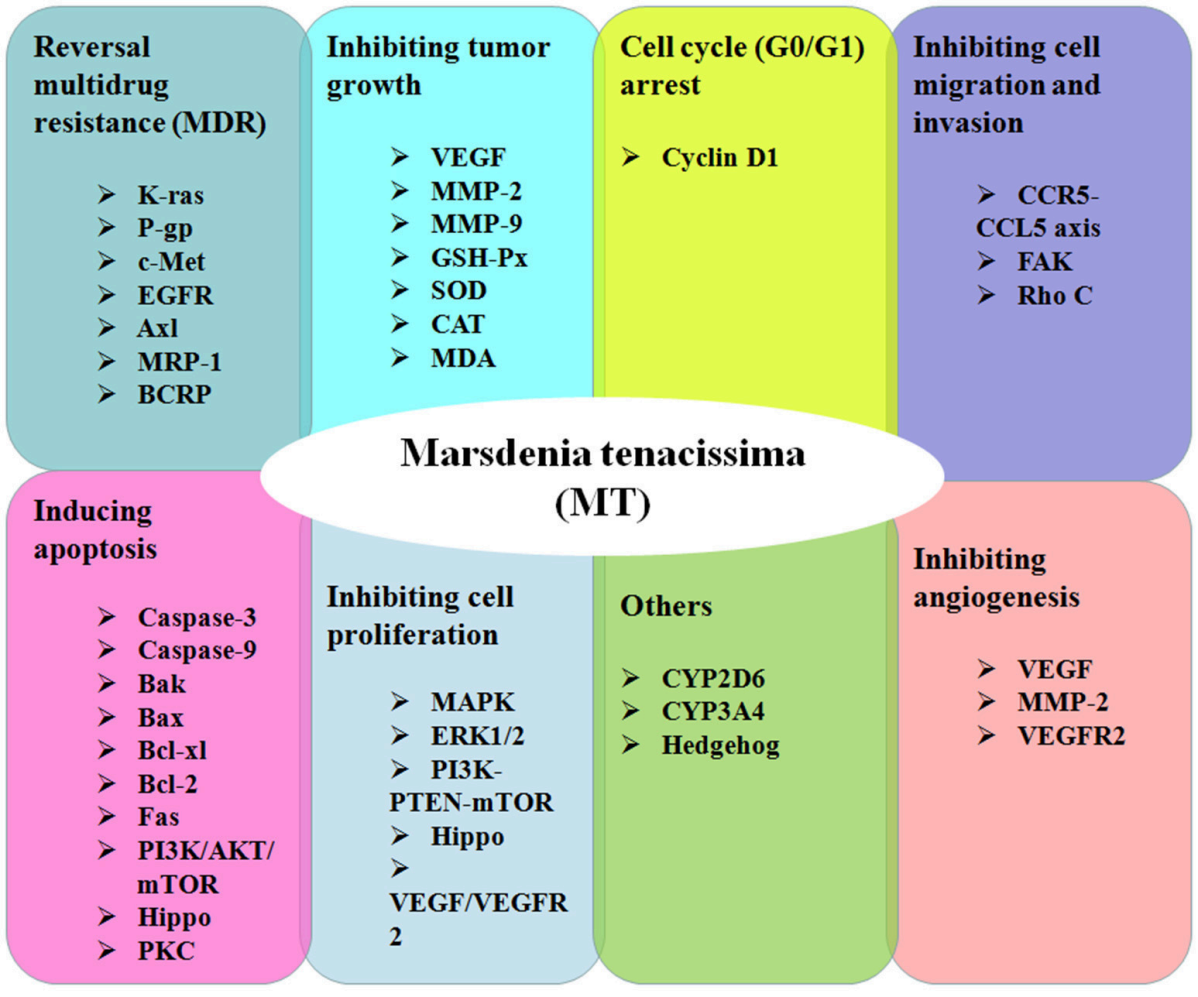
The mechanisms and biological effects of *Marsdenia tenacissima* in cancer research.

In this review, we mainly discuss the findings for the antitumor activities of MT and mechanisms relevant to antitumor effects (Figure 2, Table 1). Furthermore, as a promising natural product that may be widely used in future clinical applications, the toxic side effects and application value of MT will also be discussed.

## *Marsdenia tenacissima* effects against lung cancer

Lung cancer is one of the most frequent malignant tumors and the leading cause of cancer-related deaths in the world. Studies indicated that non-small cell lung cancer (NSCLC) accounts for ~ 85% of all lung cancer cases (35). Radiotherapy, chemotherapy, and surgical resection are the major treatments for lung cancer patients, whereas the results are often not satisfactory. In recent years, a number of traditional Chinese medicines (TCM), such as MT, has been added, as an adjuvant therapy with significant effects on the treatment of lung cancer through enhanced sensitivity of the radiotherapy and chemotherapy, decreased adverse reactions and improved quality of life (36). Results of one meta-analysis have indicated XAP injection together with chemotherapy increased the effective rate and quality of life improvement rate and reduced the toxicities of chemotherapy in relation to chemotherapy alone used in the treatment of NSCLC. It has also been shown that XAP injection could restore gefitinib sensitivity in chemoresistant lung cancer cells (37). It is well known that the epidermal growth factor receptor (EGFR) tyrosine kinase inhibitors (TKIs) are used to treat NSCLC patients clinically through blocking of intracellular receptor phosphorylation (38). However, multiple factors could result in resistance to TKIs, such as EGFR mutations, c-Met gene amplification and K-Ras mutations. The PI3K/AKT/mTOR pathway is an intracellular signaling pathway important in cell cycle regulation that is overactive in many cancers, promoting proliferation and reducing apoptosis (39). Studies have demonstrated that MTE administration could restore the gefitinib sensitivity in gefitinib-resistant NSCLC cells H460 and H1975 *in vivo* and *in vitro* through downregulating the ERK1/2, c-Met, and PI3K/AKT/mTOR pathways (19, 20). Moreover, MTE could also improve gefitinib efficacy in NSCLC cells regardless of EGFR status (21). Tenacigenin D, a compound identified from MT, strengthened the activity of erlotinib and gefitinib in two resistant NSCLC cell lines H292 and H460, through reversing both K-Ras mutation and P-glycoprotein (P-gp) overexpression mediated multidrug resistance (MDR) mechanisms (18). It was well known that bypass pathways driven by Axl and c-Met share the same downstream signaling cascade as EGFR. Research has indicated that MTE could restore erlotinib and gefitinib efficacy in the resistant NSCLC cell line HCC827/ER, with Axl and c-Met activation *in vitro* and *in vivo*, through suppression of EGFR downstream molecules (17). In addition to the effects on these signaling pathways, MTE could inhibit gefitinib metabolism by disturbing the major CYP enzyme activities of CYP2D6 and CYP3A4, lowering gefitinib metabolic clearance and increasing gefitinib concentration in the HepG2 human hepatoma cell line (26). Metastasis is the leading cause of death in cancer patients. The CCR5-CCL5 axis has been shown to be a potential markers for metastatic cancer, and their interaction leads to increased cancer cell invasion (40). XAP injection could inhibit migration and invasion in A549 lung cancer cells by downregulating the CCR5-CCL5 signaling pathway (41).

## *Marsdenia tenacissima* effects against hepatic carcinoma

Hepatic carcinoma (HCC) is the fifth most common cancer, ranking as the third most common cause of cancer-related death worldwide (42). It is difficult to remove surgically as it is typically diagnosed at an advanced stage, accompanied by frequent intrahepatic spread and extrahepatic metastasis (43). Studies have shown that MT has promising anti-hepatoma effects when used alone or combined with chemotherapeutics. One water-soluble polysaccharide, *Marsdenia tenacissima* polysaccharide (MTP), can improve immune function in normal mice and inhibit tumor growth in H22 hepatic carcinoma cells in tumor-bearing mice by significantly enhancing the activities of certain substances, such as GSH-Px, SOD, and CAT (11). YAP, a direct downstream effector of the tumor suppressive Hippo pathway, has been reported to modulate the downstream target genes that inhibit apoptosis and mediate cell proliferation (44). Acting as a potential tumor promoter, YAP overexpression has been connected with the progress of various tumors (45). Research has found that C21 steroids isolated from MTE can effectively induce apoptosis and inhibit the proliferation of HCC cells through inhibition of the PI3K/AKT/mTOR and YAP pathways together (28).

## *Marsdenia tenacissima* effects against hematological malignancy

Leukemia, also known as blood cancer, is a kind of malignant tumor in the hematopoietic system. Generally, it can be divided into several types, such as acute lymphoblastic leukemia (46), acute myeloid leukemia (AML), and chronic myelocytic leukemia (CML) (47). Recent studies have demonstrated that the crude ethanolic extract of *Marsdenia tenacissima* (CME) exhibited strong cytotoxicity against CML cell lines K562 *in vitro* and *in vivo*, and this cytotoxicity was related to inducing cell cycle (G0/G1) arrest through upregulating cyclin D1 and cell apoptosis through upregulating pro-apoptosis proteins caspase-3, caspase-9 and Bax (3). Specifically, tenacissoside C, another compound isolated from MT, had strong suppressive effects on CML cell line K562 cell-bearing nude mice through promoting G0/G1 cell cycle arrest and apoptosis (25). Similarly, another report showed that MTE also suppressed cell proliferation and induced apoptosis in Jurkat leukemia cells by inactivating the PI3K/AKT/mTOR signaling pathway (22).

Lymphoma is a large group of lymphoid hematopoietic malignancies including Hodgkin's lymphoma and non-Hodgkin's lymphoma. With advances in understanding the biology and genetics of lymphoma, many new agents, including natural agents, are being used in the treatment of lymphoma (48). According to the studies, vascular endothelial growth factor (VEGF) is one of the most effective and specific facilitators of angiogenesis and it forms new blood vessels by stimulating endothelial cell (EC) proliferation. Matrix metalloproteinases (MMPs), a family of at least 24 zinc-dependent endopeptidases that degrade the basement membrane and all protein components of the extracellular matrix, are presumed to play an important role in angiogenesis. MMP-2 and MMP-9 play essential roles in cancer cell metastasis and invasion and are abundantly expressed in diverse malignant cells (49). One study revealed that MTE inhibited tumor growth and decreased angiogenesis in A20 lymphoma cells through reduction of the expression of VEGF, MMP-2, and MMP-9 in the serum (23). Meanwhile, MT, with its major constituents tenacigenoside A (TGTA) and 11α-O-benzoyl-12β-O-acetyltenacigenin B (TGTB), could significantly induce apoptosis in Raji lymphoma cells (24).

## *Marsdenia tenacissima* effects against other tumors

MT also has cytotoxic effects on other tumors other than those described above. For example, Fas cell surface death receptor (Fas), which belongs to the tumor necrosis factor receptor super family, is a trans-membrane protein that is widely expressed on the cytoplasmic membrane. After interacting with its ligand, Fas can promote the apoptotic signal in cells (46, 50). MTE was able to enhance the curative effect of doxorubicin chemotherapy in MG63 osteosarcoma cells, through upregulation of Fas expression in tumor cells (30). MAPK (mitogen-activated protein kinase) signaling pathways play a leading role during transfer of an extracellular signal into the cytoplasm and nucleus, inducing a variety of cellular biological functions, such as cell immunity, development, and apoptosis (51–53). Studies showed that MTE had a good inhibitory influence on esophageal cancer cells KYSE150 and Eca-109 through inhibiting the MAPK signal transduction pathway (9).

## Isolation and structural characterization of compounds from MT

In order to isolate novel products from MT, particularly those with potential bioactivity, it is important to firstly perform a dereplication process (54), distinguishing the novel compounds from previously reported compounds. Techniques capable of quickly and accurately identifying previously isolated compounds are therefore of great value. One approach to dereplication is to use high-performance liquid chromatography (HPLC) coupled to electrospray ionization-multiple stage tandem mass spectrometry (ESI-MS/MS) to efficiently identify known or novel pregnane glycosides from MT extracts (55). Recently, a sodium and ammonium adduct ion-targeted product ion scans (PIS) system has been developed to elucidate the structural difference of novel POPs in herbal samples (56). Wang et al. developed a novel homologs prediction strategy for effective discovery of unreported chemical components from TM extracts (57). In addition, employing a capillary electrophoresis method, Zhao et al. could separate the reported components from the TM extracts quickly and accurately (18). Clearly, all these methods can readily provide information useful for dereplication and preparation of complex mixtures isolated from MT.

Based on these dereplication measures, several systematic phytochemical studies have been performed to elucidate the C21 steroid derivatives identified from MT. From the findings of Pang's group, 23 C21 steroidal glycosides (marstenacisside C1–C10, D1–D7, and E1–E6), and four C21 steroids, 11a,12b-O-ditigloyl-tenacigenin C, 11a-O-benzoyl-12b-O-tigloyltenacigenin C, 11a-O-tigloyl-12b-O-benzoyl-tenacigenin C, and 11a-O-tigloyl-12b-O-benzoyl-marsdenin, were obtained from the 95% alcoholic extract of the MT roots. The chemical structures analysis by spectroscopic techniques, such as NMR spectroscopy, indicated a C_21_ steroidal skeleton for all these 27 compounds. Further ^13^C-NMR data suggested that these compounds had the same sugar moieties located at the C-3 hydroxyl group (58). This same group has also isolated 16 new POP glycosides, marstenacissides A1–A7 and marstenacissides B1–B9, from MT roots. All the compounds are steroidal glycosides with 2-deoxysugar moieties. And the spectrographic analyses of NMR suggested that these 16 compounds have the structural patterns of C21-steroid diester derivatives with the oligosaccharide sugar moiety consisting three or four units (34). From the MT stems, Xia et al. have identified five new pregnane glycosides, namely marstenacissides E, F, G, H, and I. Though differed in the sugar moiety, these 5 compounds had the same aglycon, which was a pregnane highly oxidized at C-3, C-8, C-12, C-14, C-17, and C-20 (59). Apart from these constituent above-mentioned, to data, much more other extracts with the similar steroidal structures have been isolated from different research groups (55, 60). Furthermore, these newly-reported compounds have been proved to have potential tumor-killing effects (18, 61) or antiviral functions (62). Taken together, these findings mainly study on the isolation and structure features of POP glycosides from MT, and their biological activities, as an aid to understanding the constituents of this herb medicine, which will benefit the development of this medicine as well as its preparation.

## Safety and efficacy

MT extracts have long been applied to anti-tumor therapy as an adjuvant medicine. As potential anti-tumor agents, MT extracts have a broader spectrum of activity against a large number of tumors both *in vivo* and *in vitro*. As to the significant cytotoxicity of MT extracts, evaluating their clinical safety is very important. A recent study indicated that MTE induced aging and cytotoxicity in erythrocytes in a dose-depend manner through increasing calcium and ROS levels, elevating the ratio of erythrocyte shrinking and fragmentation (15). This result implied the potential damage on cancer patients' circulating erythrocytes when MTE was used as an anti-tumor medicine. However, in the experiment on albino rats to evaluate the acute and subacute toxicity induced by MTE leaves, the result revealed that MTE leaves caused no obvious celluar toxicity (63). In general, the study give a brief description of the safety of MT. Obviously, more surveys on the clinical applications of MT are required to evaluate the safety and effectiveness. Above all, these observations will help direct treatment while avoiding the side effects of MT.

From another perspective, the accurate identification and pharmacokinetics evaluation of herb medicine extracts is highly necessary to ensure their safety and efficacy (64). Considering that structures of steroidal glycosides are similar, evaluating the pharmacokinetic properties of the MT extracts will be useful to ensure the efficacy and safety of this herbal medicine in medicine markets and clinical application. A sensitive and specific liquid chromatography–ESI-MS/MS assay, LC–ESI-MS/MS, was developed to quantify the plasma concentration of Tenacissoside A, the major active constituent of MT, in rats after intravenous route at 0.1 mg/kg or intragastric administration at 1 mg/kg. The concentration range validated by this method was 1–250 ng/mL for Tenacissoside A (65). Another more sensitive analytical LC-MS/MS method with one-step protein precipitation was proposed to simultaneously determine the plasma concentration of two steroidal glycosides, tenacissoside H, and tenacissoside I, in rat after oral gavage. The lower limits of quantification was 0.9 ng/mL for these two MT extracts (66). Recently, Yu et al. developed a new evaluation model combining DNA barcoding techniques with thin-layer chromatography (TLC) and HPLC. This effective strategy could facilitate pharmacologist to identify and quantify of MT extracts in the medicine market (67). In addition, given biotransformation *in vivo* may influence the chemical structure of a compound and change its activity (68), identification of metabolic characteristics could provide valuable information for further understanding the anti-tumor activity of MT. Using the ultra-HPLC coupled with high-resolution MS assay, Zhao et al. identified the metabolic profile of C-21 steroids from MT for the first time. The fragmentation data from human liver microsomes indicated that hydroxylation reactions were the major metabolic pathway of Tenacissoside H and Tenacissoside I, whereas the metabolic pathway of Tenacigenin B mainly involved dehydrogenation reactions. Meanwhile, about 14 novel metabolites yielded from these C-21 steroids was characterized and identified precisely and quickly (33).

## Discussion

MT is a well-known medicinal plant possessing antitumor effects. For example, ATP-binding cassette (ABC) transporters could remove substrates from the cell against a concentration gradient, leading to MDR (69). POPs, the main constituents of MT, can inhibit different ATP-binding cassette (ABC) transporters, including P-gp, multidrug resistance associated protein-1 (MRP1) and breast cancer resistance protein (BCRP or ABCG2), contributing to MDR reversal as well as improving effectiveness of traditional anticancer drugs in tumors (10). More specifically, tenacissimoside A and 11α-O-benzoyl-12β-O-acetyltenacigenin B, two compounds identified from MT, reversed P-gp mediated MDR in HepG2/Dox cells and enhanced the sensitivity of antitumor drugs, such as vinblastine, paclitaxel, doxorubicin, and puromycin (29). It is well known that VEGF is vital in regulating angiogenesis mediated through VEGF receptor (VEGFR), a tyrosine kinase receptor expressed at high levels in cancerous and endothelial cells (70, 71). In a recent report, MTE was able to suppress tumor angiogenesis *in vivo* and *in vitro* through decreasing VEGF-A and VEGFR-2 expression in human umbilical vein endothelial cells (HUVECs) and VEGF-A expression in HepG2 (13). Protein kinase C (PKC), which belongs to the serine/threonine kinases family and has at least 10 isoforms, also plays an essential role in the regulation of angiogenesis. P53 is a tumor inhibitor that induces cell apoptosis. MTE can accelerate apoptosis by the PKCδ-inducing p53-dependent mitochondrial pathway and inhibit proliferation through reducing CCL-2-mediated VEGF/VEGFR2 interactions in HUVECs (27). Furthermore, the Hedgehog signaling pathway plays prominent roles in the tumorigenesis and developmental processes of various types of cancer and regulates the self-renewal and proliferation of cancer stem cells. Steroidal aglycones, such as tenacigenin A, 12-O-tigloyltenacigenin A, 12-O-benzoyl-tenacigenin A, 11-O-tigloyl-12-O-acetyltenacigenin B, 11-O-tigloyl-12-O-tigloyltenacigenin B, and 11-O-acetyl-12-O-tigloyltenacigenin B, which are manufactured from MT, suppressed the Hedgehog signaling pathway in Shh-LIGHT2 cells (72). Total aglycones from MT Caulis (ETA) exhibit the ability to strengthen the antitumor activity of paclitaxel both *in vivo* and *in vitro* as a chemo-sensitizer (31). All of these findings implied that MT may have other unknown functions requiring further study for additional clinical uses.

In addition, POP glycosides are the major bioactive constituents of MT. Thus, understanding the biosynthesis of steroidal derivatives, especially pregnane and their glycosides, is very important for marker-assisted breeding of the plant. A recent study showed that a great number of gene encoding enzymes related to pregnane and cholestenol backbone biosynthesis were discovered, including EBP (cholestenol Δ-isomerase), DHCR24 (Δ24-sterol reductase), DHCR7 (7-dehydrocholesterol reductase), LAS (lanosterol synthase), 4-MSO (C4-methylsterol oxidase), 14-SDM(sterol-14α-demethylase), 14SR (Δ14-sterol reductase), 3β-HSD (3β-hydroxysteroiddehydrogenase), SC5DL (sterol C5 desaturase/lathosterol oxidase), and 5β-POR (progesterone 5β-reductase), as well as several candidate genes CAS, SQS, SQLE, 3β-HSD, 4-MSO, and 5β-POR (8). Those results could promote functional studies to produce an abundance of these compounds for cancer treatment. It is well known that biotransformation *in vivo* or *in vitro* can impact the structure of a compound and change its activity. Therefore, understanding their metabolites and fragmentation behaviors is helpful for optimal utilization. Research has indicated that tenacissoside I and tenacissoside H, two compounds isolated from MT, were mainly metabolized through hydroxylation reactions, while dehydrogenation reactions were the principal metabolic pathway for tenacigenin B (33). As a promising TCM, further study can provide us with valuable new information for understanding the activity of MT.

*Marsdenia condurango* (Family Asclepiadaceae), which is similar to MT and commonly called condurango, primarily comes from the northwestern part of South America. Condurango contains pregnane glycosides and shows antitumor effects. For example, the ethanolic extract of *Marsdenia condurango* could relieve BaP-induced lung cancer in rats through a caspase-3-dependent pathway to induce apoptosis (73). Condurangogenin A, an isolated active ingredient, has anticancer effects against several lung cancer cell types *in vitro* by DNA damage-induced apoptosis and p21/p53 mediated cell cycle regulation (74). Moreover, condurango glycoside-rich components (CGS) exhibited suppression of cell-proliferation in lung cancer, *in vivo* and *in vitro*, via ROS-mediated caspase-3 dependent apoptosis and DNA damage-induced cell cycle arrest (75). Condurango 30C could induce epigenetic modification in lung cancer through regulation of DNA hypermethylation (76). *Marsdenia condurango* and MT belong to the same family and reveal similar effects through various mechanisms. As a result, these findings could provide a clue to finding more active substances for enriching our clinical drug species.

## Conclusions

In conclusion, searching for active ingredients from plants to treat diseases is a promising research direction for creating novel drugs both at home and abroad. MT has significant antitumor effects as demonstrated by clinical practice and has significant clinical potential due to its as yet undiscovered activities. Nevertheless, investigations into the mechanisms of antitumor activity of MT have only just begun, and clinical research is lacking the high-level of evidence required for evidence-based medicine. Thus, to further clarify the effectiveness and mechanisms of its antitumor effects, conduct a multicenter randomized controlled trial in clinical settings will be the direction of future research.

## Author contributions

YY, ZG, and ZX conception and design. XW, SZ, LQ, XY, XC, JW, XR, and YZ wrote the manuscript. YY and ZX revised the manuscript. All authors reviewed and approved the final version of the manuscript.

### Conflict of interest statement

The authors declare that the research was conducted in the absence of any commercial or financial relationships that could be construed as a potential conflict of interest.
